# Integrative analysis of scRNA-seq and bulk RNA-seq with machine learning develops a nucleotide metabolism–based prognostic model for ccRCC and reveals the function of IFI30

**DOI:** 10.3389/fonc.2026.1881692

**Published:** 2026-08-14

**Authors:** Qiao Lyu, Ping Li, ZhenXiong Ye, JiaHui Chen, Feng Luo, QiYu Zhong, GuoHao Wu, HanDa Zheng, JianSheng Xiao, DongMing Ye, LiJun Qu

**Affiliations:** 1 Department of Urology, The Sixth Affiliated Hospital of Jinan University (Dongguan Eastern Central Hospital), Dongguan, Guangdong, China; 2 Department of Urology, The First Affiliated Hospital of Jinan University, Guangzhou, Guangdong, China

**Keywords:** clear cell renal cell carcinoma, IFI30, machine learning algorithms, nucleotide metabolism, scRNA-seq

## Abstract

**Objectives:**

Nucleotide metabolism in clear cell renal cell carcinoma (ccRCC) remains understudied. Elucidating its heterogeneous characteristics and key genes may provide new insights for prognostic assessment and targeted therapy.

**Methods:**

By integrating scRNA-seq with bulk RNA-seq data, we first screened for nucleotide metabolism-related genes (NMRGs) using five machine learning algorithms. NMRGs signature was then constructed using 101 machine learning algorithms to optimize clinical prognosis assessment. The selected NMRGs were subjected to GO functional annotation and KEGG pathway enrichment analysis. Finally, the functional role of the key gene IFI30 was validated through both *in vitro* and *in vivo* experiments.

**Results:**

The NMRG signature constructed using 101 machine learning algorithms outperformed traditional clinical parameters and 122 published models across multiple independent cohorts. High-risk patients exhibited significantly worse overall survival. Enrichment analysis showed that IFI30 and its associated genes were significantly enriched in nucleotide metabolism pathways. Immunohistochemistry confirmed high IFI30 expression in ccRCC tumor tissues. Functional assays demonstrated that IFI30 knockdown suppressed ccRCC cell proliferation, migration, invasion, and tumorigenicity, while inducing apoptosis. In addition, IFI30 knockdown downregulated PRPS1/2, a key rate-limiting enzyme in nucleotide synthesis, and nucleotide rescue experiments reversed the phenotypic suppression, confirming that IFI30 promotes ccRCC progression through nucleotide metabolism.

**Conclusion:**

This study developed an NMRGs signature that outperforms existing models and, for the first time, reveals that IFI30 promotes ccRCC malignant progression by regulating nucleotide metabolism. These findings provide a new theoretical basis for prognostic assessment and metabolism-targeted therapy in ccRCC.

## 1 Introduction

Renal cell carcinoma (RCC) is one of the most aggressive and lethal tumors of the urinary system. Over the past two decades, the incidence and mortality rates of RCC have increased annually worldwide (1). Early-stage RCC lacks specific clinical manifestations, consequently, approximately 30% of patients are diagnosed at an advanced stage, with possible metastasis already present (2). For advanced renal cell carcinoma (RCC), traditional chemotherapy and radiation therapy are both ineffective. In contrast, targeted therapy and immune checkpoint inhibitors have become the standard first-line treatments. Although these therapies demonstrate significant initial efficacy, most patients eventually develop resistance, leading to subsequent disease progression or metastasis (3, 4). Clear cell renal cell carcinoma (ccRCC) is the major histological subtype of RCC. Previous studies have associated its development with obesity, smoking, hypertension, and environmental factors; however, the exact molecular mechanisms underlying ccRCC tumorigenesis remain unclear. Timely diagnosis and treatment guided by reliable biomarkers could significantly improve patient survival rates (5).

A fundamental characteristic of tumor cells is metabolic alteration. This phenomenon is referred to as metabolic reprogramming (6). Nucleotide metabolism plays a critical role in the progression of various tumors. Unlike carbohydrate, lipid, or amino acid metabolism, which primarily support energy supply, membrane structure synthesis, or protein translation, nucleotide metabolism directly provides essential building blocks for DNA replication and repair, RNA synthesis, and signal transduction (ATP, GTP, CTP, UTP, and dNTPs) (7–9). During tumor progression, this metabolic pathway is upregulated to support rapid proliferation and can induce genomic instability, thereby driving key carcinogenic processes, including sustained proliferation, chemotherapy resistance, immune evasion, and metastasis (10, 11). Therefore, targeting nucleotide metabolism may not only suppress tumor proliferation but also reshape the tumor-immune microenvironment, potentially affecting the efficacy of targeted therapy (12).

Previous studies have begun to elucidate nucleotide metabolism in ccRCC. Early gene expression profiling of renal cell carcinoma samples revealed an association between nucleotide metabolism and patient survival (13). Recent studies have further uncovered its link to sunitinib resistance and identified novel targets such as LEDGF, TRIM28 (13–15). Nevertheless, current research remains markedly limited. Most efforts focus on individual metabolic enzymes or single pathways, failing to systematically characterize the heterogeneity of nucleotide metabolism in ccRCC. Consequently, systematic and in-depth investigations into nucleotide metabolism and its prognostic value in ccRCC are still lacking. Single-cell transcriptomics enables high-resolution, comprehensive profiling of diverse cell types within the tumor microenvironment. Its integration with bulk RNA-seq offers the dual advantage of enabling biological discovery at single-cell resolution while supporting clinical translation via bulk-based prognostic models. Meanwhile, as a key branch of artificial intelligence, machine learning (ML) can construct reliable predictive models based on large-scale data, Using 101 ML overcomes the selection bias associated with any single algorithm and achieves accurate prediction (16).

This study systematically constructed a nucleotide metabolic signature of ccRCC by integrating single-cell RNA-seq (scRNA-seq) and bulk RNA-seq data. Using bioinformatics analysis and ML, we established a robust prognostic model and evaluated its clinical utility. Additionally, we identified IFI30 as a novel therapeutic target. Through *in vitro* and *in vivo* experiments, we further elucidated the regulatory role of IFI30 in nucleotide metabolism in ccRCC, providing a theoretical foundation for the development of targeted therapeutic strategies.

## 2 Methods

### 2.1 Sources of bulk RNA-seq data

Transcriptomic data and corresponding clinical information for ccRCC were obtained from The Cancer Genome Atlas (TCGA) database. RNA-seq count data were downloaded, converted to transcripts per million (TPM), and log2-transformed using log2(TPM + 1) to reduce data skewness. The GSE29609, GSE167573, E-MTAB-1980, and CPTAC-ccRCC datasets were retrieved from public repositories and used as external validation cohorts.

For microarray datasets, quantile normalization was first performed using the normalizeBetweenArrays function in the limma package to reduce technical differences among samples (17). When multiple bulk transcriptomic cohorts were merged for downstream analysis, the dataset or cohort source was defined as the batch variable, representing the cohort/platform origin rather than individual patient samples. Batch effects across cohorts/platforms were adjusted using the ComBat algorithm implemented in the sva package. Principal component analysis (PCA) was performed before and after ComBat correction using the merged bulk transcriptomic expression matrix to evaluate the effectiveness of batch-effect removal. For external validation, each independent cohort, including GSE29609, GSE167573, E-MTAB-1980, and CPTAC-ccRCC, was normalized and analyzed separately to avoid introducing additional cross-platform bias. All expression matrices were subsequently log2-transformed before downstream analysis (18, 19).

### 2.2 Single-cell RNA sequencing analysis

scRNA-seq data were obtained from the Gene Expression Omnibus (GEO) database and processed using Seurat and Harmony in R (20). Raw count matrices were imported to generate Seurat objects, and all samples were merged after assigning unique cell barcodes. Quality control was performed based on the number of detected genes, UMI counts, mitochondrial gene percentage, ribosomal gene percentage, and hemoglobin gene percentage. Cells with 200–10,000 detected genes, more than 1,000 UMI counts, mitochondrial gene percentage <20%, and ribosomal gene percentage <20% were retained. Genes expressed in more than three cells were kept for downstream analysis. Hemoglobin gene percentage was calculated and visualized as an additional quality-control metric but was not used as a final filtering criterion. The raw barcodes/cells before quality control and the retained cells after quality control for each sample are summarized in **Supplementary Table 1**.

Data were normalized using NormalizeData, and highly variable genes were identified with FindVariableFeatures (21). Scaling was performed with ScaleData, followed by PCA via RunPCA. To eliminate batch effects across samples, Harmony was applied for integration. A cell proximity network was constructed using the top 30 PCs, and clustering was performed using the Louvain algorithm (22). Cells were visualized using UMAP (23).

### 2.3 Identification of nucleotide metabolism-related genes

To systematically identify nucleotide metabolism-related genes (NMRGs), we searched the GeneCards database using the keyword “nucleotide metabolism”. GeneCards integrates multiple sources of gene annotation and provides a relevance score to reflect the strength of association between a gene and the queried biological term. To reduce background noise from weakly associated genes and retain genes with relatively strong functional relevance to nucleotide metabolism, genes with a relevance score > 10 were selected as candidate NMRGs. This threshold was applied before downstream single-cell metabolic scoring, differential expression analysis, and machine learning-based feature screening. Therefore, the final NMRG candidates were not determined solely by the GeneCards score, but were further refined through single-cell expression analysis and multiple machine learning algorithms.

### 2.4 ssGSEA scoring and differential expression analysis

To assess nucleotide metabolic pathway activity at the single-cell level, we performed single-sample gene set enrichment analysis (ssGSEA) to calculate a nucleotide metabolism score for each cell using the GSVA R package (24). Cells were divided into high- and low-score groups based on the median score. Differential expression analysis between the two groups was then performed using the limma package, with thresholds of |log2 fold change| > 0.5 and P < 0.05. The resulting differentially expressed genes (DEGs) were used for subsequent machine learning analysis.

### 2.5 Identification of key genes using machine learning

To further identify key genes associated with nucleotide metabolism, five machine-learning algorithms were applied to the 136 differentially expressed genes between the high- and low-nucleotide metabolism score groups. Before model training, expression values of the filtered genes were extracted from the single-cell expression matrix and combined with nucleotide metabolism group labels. A fixed random seed of 123 was set before the feature-selection workflow to improve reproducibility.

Boruta feature selection was performed using the Boruta R package. The maximum number of Boruta runs was set to 500, and tentative features were further processed using TentativeRoughFix; only confirmed genes were retained. XGBoost was performed using the xgboost R package with objective = “binary:logistic” and nrounds = 50, and genes were ranked according to feature importance. LASSO regression was conducted using the glmnet R package with family = “binomial” and alpha = 1; genes with non-zero coefficients at lambda.min were retained. Random forest analysis was performed using the randomForest R package with ntree = 500 and importance = TRUE, and genes were ranked according to variable importance. Pamr analysis was performed using the pamr R package, and genes were selected using a nearest shrunken centroid threshold of 2 (24). The selected gene lists generated by the five algorithms were exported and summarized in **Supplementary Table 3** to improve the traceability of the feature-selection process. The overlapping genes among the five algorithms were identified using a Venn diagram and retained as candidate feature genes for subsequent prognostic model construction.

### 2.6 Construction of NMRGs signature

In the TCGA-ccRCC cohort, prognostic models were constructed based on the seven hub genes using a machine-learning framework composed of 101 algorithm combinations. The candidate algorithms included SuperPC, random survival forest (RSF), CoxBoost, LassoCox, gradient boosting machine (GBM), elastic net, ridge regression and other survival-learning methods. The main R packages used for model construction included superpc for SuperPC, randomForestSRC for RSF, CoxBoost for CoxBoost, glmnet for LassoCox, gbm for GBM, and survival for Cox regression modeling (25). For algorithms requiring parameter tuning, cross-validation was used to select the optimal parameters. Specifically, cross-validation was used for threshold selection in SuperPC, boosting step selection in CoxBoost, and penalty parameter selection in LassoCox. For RSF and GBM, model performance was evaluated using survival prediction performance and C-index. A fixed random seed was set before model training to improve reproducibility.

The optimal model was not selected solely according to the C-index in the training cohort. Instead, model performance was comprehensively evaluated across the TCGA training cohort and four independent validation cohorts, including GSE29609, GSE167573, E-MTAB-1980, and CPTAC-ccRCC. The final NMRGs signature was selected based on overall predictive performance, cross-cohort stability, Kaplan–Meier survival stratification, and time-dependent ROC performance. This strategy was used to reduce the risk of selecting a model that performed well only in the training cohort.

### 2.7 External cohort validation

To validate the NMRGs signature, we tested it across multiple independent cohorts, including GSE29609, GSE167573, E-MTAB-1980, and CPTAC-ccRCC. Kaplan–Meier analysis with the log-rank test was used to compare overall survival between high- and low-risk patients, and hazard ratios (HRs) with 95% confidence intervals (CIs) were calculated (26). Time-dependent ROC curves were generated using the timeROC package to assess the model’s predictive accuracy for 1-, 3-, and 5-year overall survival (26).

To further compare the prognostic performance of the NMRGs signature with previously published signatures, 122 published prognostic models were collected from the literature. For each published model, the risk score was recalculated as the sum of gene expression values weighted by the corresponding reported coefficients. Only genes available in the analyzed expression matrix were included for recalculation. C-index values were calculated using Cox proportional hazards models implemented in the survival R package, and pairwise C-index comparisons between the NMRGs signature and published models were performed using the compareC R package. Detailed information on the 122 published models, including PMID, model type, gene composition, coefficients, and software/package versions used for recalculation, is provided in **Supplementary Table 4**.

### 2.8 Metabolic pathway analysis

To investigate the relationship between the NMRGs signature and metabolic characteristics in ccRCC, we used the Kyoto Encyclopedia of Genes and Genomes (KEGG) database and ssGSEA to calculate activity scores for each metabolic pathway per sample. Patients were divided into high- and low-risk groups based on NMRGs signature, and differences in nucleotide metabolism between the two groups were compared.

To explore the potential function of IFI30, we screened IFI30-associated genes from the LinkedOmics database (|Statistic| ≥ 0.30, FDR < 0.05). Gene Ontology (GO) enrichment analysis (Biological Process (BP), Cellular Component (CC), Molecular Function (MF)) was performed using clusterProfiler, with Benjamini-Hochberg correction (adjusted p < 0.05). GSEA was also conducted using the gseKEGG to identify pathways associated with IFI30 expression, and pathways were ranked by normalized enrichment score (NES).

### 2.9 Immunohistochemistry of human tissue samples

Surgical specimens were collected from 16 patients with ccRCC at the First Affiliated Hospital of Jinan University (Guangdong, China), of which 3 cases were randomly selected for immunohistochemical analysis. Tumor and matched peritumoral tissues from three randomly selected patients were formalin-fixed, paraffin-embedded, and sectioned. Sections were deparaffinized, rehydrated, and subjected to antigen retrieval in citrate buffer (pH 6.0) using a pressure cooker. Endogenous peroxidase was blocked with 0.3% H_2_O_2_, and non-specific sites were blocked with 10% sheep serum. Sections were incubated overnight at 4 °C with anti-IFI30 antibody (Proteintech, Wuhan, China; 1:200), then with secondary antibody (Proteintech, Wuhan, China; 1:1000) at room temperature. Staining was developed with DAB, followed by hematoxylin counterstaining, dehydration, clearing, mounting, and air-drying. Images were captured using a light microscope.

### 2.10 Cell culture and transfection conditions

The ccRCC cell lines 786-O and Caki-1 were purchased from the American Type Culture Collection (ATCC, Manassas, VA, USA) and authenticated by STR profiling. Cells were cultured in RPMI-1640 medium (Gibco, ThermoFisher, Waltham, MA, USA) supplemented with 10% fetal bovine serum (FBS, Procell, Wuhan, China) in a humidified 5% CO_2_ incubator at 37 °C.

To knock down IFI30 and establish stable IFI30-knockdown ccRCC cell lines, shRNA sequences targeting IFI30 and a negative control (shNC) were designed and cloned into a lentiviral vector (IGE Biotechnology Co., Ltd, Guangzhou, China).786-O and Caki-1 cells were seeded at 5×10^4^ cells/well in 6-well plates and transduced with lentiviral particles and 5μg/mL polybrene (Beyotime, Shanghai, China). Stable cells were selected with 2μg/mL puromycin (Beyotime, Shanghai, China), the minimum effective concentration determined in preliminary experiments. Sequences: shIFI30-1(5’-GCGTTAGACTTCTTTGGGAAT-3’)、shIFI30-2(5’-CTACGGAAACGCACAGGAA-3’)、shIFI30-3(5’-GTTAGACTTCTTTGGGAAT-3’)、shNC(5’-TAAGGGTTCTTCAGTATTGCG-3’).

### 2.11 qRT-PCR

Total RNA was extracted from 786-O and Caki-1 cells using TRIzol™ (Invitrogen, CA, USA). The RNA was reverse-transcribed into cDNA and amplified by qRT-PCR according to our previously reported method (27). Relative expression levels were calculated using the 2^−^ΔΔCt method with GAPDH as the internal control. Primer sequences were: IFI30 (F:5’-CCCTCTGCAAGCGTTAGACT-3’; R:5’-CAAGCGGTGCATTGGACTTC-3’), GAPDH (F: 5’- AACGGATTTGGTCGTATTG -3’; R: 5’- GGA AGATGGTGATGGGATT -3’).

### 2.12 Western blotting

Proteins were extracted from cells using RIPA buffer (Beyotime, Shanghai, China) on ice, separated by 8% SDS-PAGE, and transferred to PVDF membranes as previously described (27). Membranes were blocked with 5% non-fat milk for 40 minutes at room temperature. After washing, membranes were incubated overnight at 4 °C with primary antibodies: anti-IFI30 (Abcam, Cambridge, MA, USA; 1:1000), anti-β-actin (Beyotime, Shanghai, China; 1:2000), and anti-PRPS1/2(Abcam, Cambridge, MA, USA; 1:1000). Following primary antibody incubation, membranes were incubated with HRP-conjugated secondary antibody (Beyotime, Shanghai, China) for 1 hour at room temperature. Protein bands were visualized using an ECL kit (Beyotime, Shanghai, China). β-actin served as the loading control. Relative protein expression levels were normalized to the control group.

### 2.13 Cell counting Kit-8 assay

To assess cell viability and proliferation, 786-O, Caki-1, and IFI30-knockdown cells were seeded in 96-well plates. For nucleoside rescue experiments, 786-O cells were divided into two groups: one group was cultured in medium supplemented with a mixture of adenosine, guanosine, cytidine, uridine, and thymidine (50μM each) (28), while the control group was cultured in normal medium. Cell viability was assessed at 24, 48, and 72 hours according to our previously reported method (27).

### 2.14 Transwell assays

786-O, Caki-1, and the corresponding IFI30-knockdown cells were cultured in 6-cm dishes until reaching 90%–100% confluence. A wound was created by scratching the cell monolayer with a 10-μL pipette tip, and the cells were then washed with culture medium. Images were captured at 0, 24, and 48 hours using a Nikon phase-contrast microscope (Nikon Instruments, Florence, Italy). Wound areas were normalized to the baseline at 0 hours, and relative wound closure was calculated by normalizing to the control group.

### 2.15 Wound-healing assay

786-O, Caki-1, and the corresponding IFI30-knockdown cells were cultured in 6-cm dishes until reaching 90%–100% confluence. A wound was created by scratching the cell monolayer with a 10-μL pipette tip, and the cells were then washed with culture medium. Images were captured at 0, 24, and 48 hours using a Nikon phase-contrast microscope (Nikon Instruments, Florence, Italy). Wound areas were normalized to the baseline at 0 hours, and relative wound closure was calculated by normalizing to the control group.

### 2.16 Colony formation assay

786-O, Caki-1, and the corresponding IFI30-knockdown cells were harvested and counted. Then, 1,000 cells were seeded into 6-well plates and cultured for two weeks until colonies became visible. The colonies were washed, fixed with methanol, and stained with 1 mg/mL crystal violet (Beyotime, Shanghai, China) for 20 minutes. After imaging, the number of distinct colonies was counted.

### 2.17 Animal models

BALB/c nude mice (4 weeks old, 20 ± 2 g) were obtained from JiNan University (Guangdong, China). IFI30-knockdown or control 786-O cells were injected into the right flank. Mice were randomly assigned to three groups (n = 5 each). Tumor volumes were measured every two days using V = 0.5×L×W². After 25 days, mice were euthanized (by cervical dislocation performed by trained personnel), and tumors were excised, weighed, and measured.

### 2.18 IHC of xenograft tumors

Tumor tissues from subcutaneous xenografts in nude mice were fixed in 4% paraformaldehyde, dehydrated, cleared, and embedded in paraffin, Sections were cut at 4μm thickness. Immunohistochemical staining for Ki67 and Caspase-3 was performed following the protocol described in Section 2.9. Primary antibodies were anti-Ki67 (1:400; Proteintech, Wuhan, China) and anti-Cleaved Caspase-3 (1:200; Proteintech, Wuhan, China). After staining, representative images were captured from each group under a light microscope; five high-power fields were randomly selected per section. Semi-quantitative analysis of positive staining was performed using ImageJ software.

### 2.19 Ethical review

The use of human specimens in this study was approved by the Ethics Committee of the First Affiliated Hospital of Jinan University (Approval No. KY-2026-202). Due to the retrospective nature of the study, informed consent was waived. All experiments were performed in strict accordance with relevant guidelines and regulations.

BALB/c nude mice were obtained from the Animal Experimentation Centre of Jinan University. All animal procedures were approved by the Laboratory Animal Centre of Jinan University (Approval No. 20250316-07).

### 2.20 Statistical analysis

All data processing and statistical analyses were performed using R software (version 4.4.1), with the exception of two-way ANOVA which was conducted using GraphPad Prism (version 10.2.3). Results are presented as mean ± standard deviation (SD). Differences between two groups were analyzed using the unpaired Student’s t-test, while multiple group comparisons were assessed by two-way ANOVA. Tumor volume differences were evaluated using the nonparametric Mann-Whitney U test. Statistical significance was set at p < 0.05.

## 3 Results

### 3.1 Construction of single-cell Atlas and cell type identification

**Figure 1:**
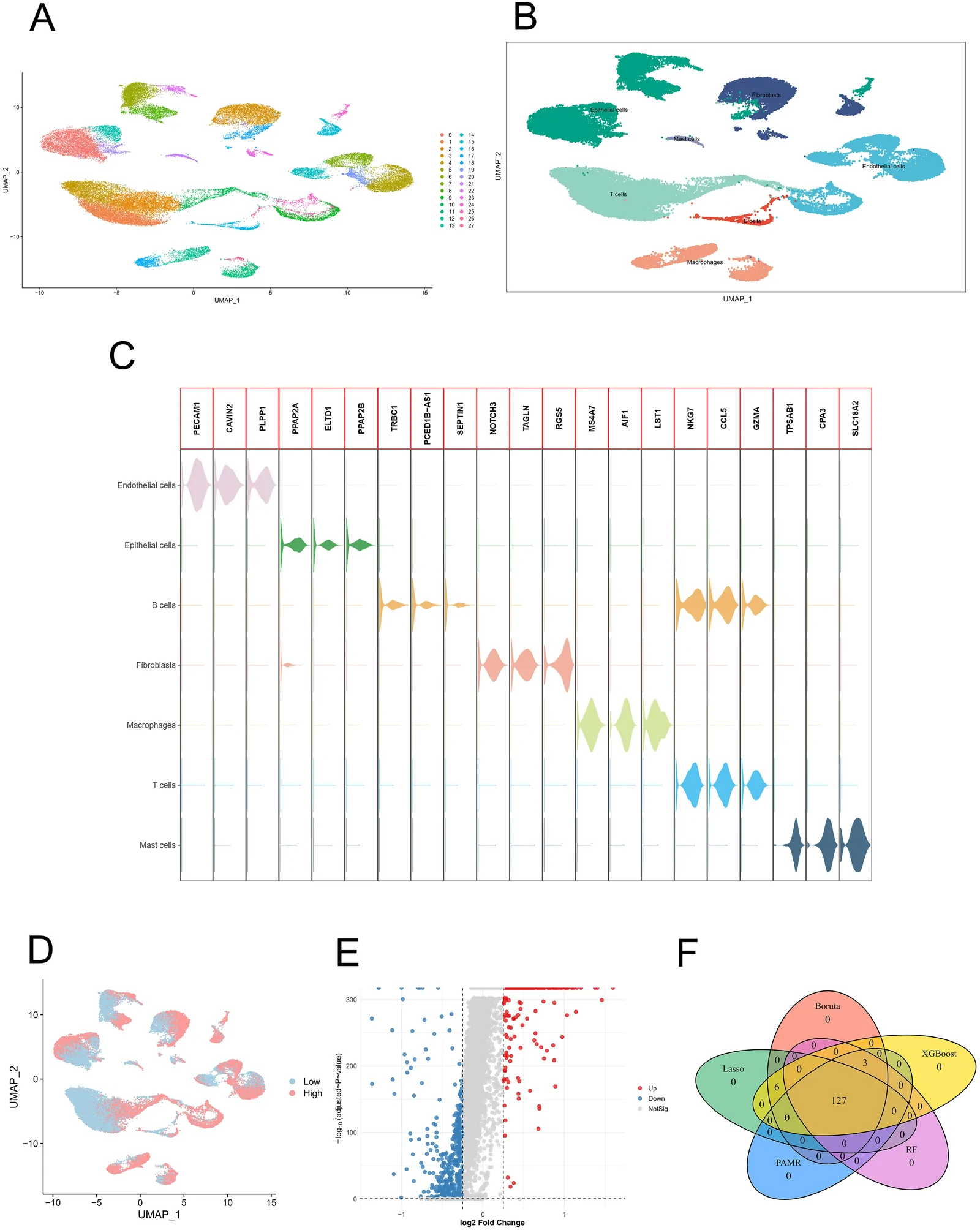
Identification of NMRGs at the single-cell level. **(A)** t-SNE plots of ccRCC and tumour microenvironment cells. **(B)** t-SNE plots showing seven major cell types. **(C)** Expression of cell-type-specific marker genes. **(D)** UMAP plot of nucleotide metabolism scores (NMRGs scores). **(E)** Volcano plot of differentially expressed genes between high- and low-score groups. **(F)** Venn diagram of genes identified by ML algorithms.

To analyze the cellular composition and molecular characteristics of ccRCC, we integrated multiple public scRNA-seq datasets and visualized all cells using UMAP. The results showed that cells formed multiple distinct clusters in the UMAP space (**Figure 1A**). Cell types were annotated based on the expression patterns of signature genes. The dataset comprised multiple cell types, including epithelial cells, T cells, B cells, macrophages, endothelial cells, fibroblasts, and mast cells (**Figure 1B**).

To verify the accuracy of cell annotations, we further analyzed the expression profiles of classic marker genes for each cell population. Different cell types exhibited distinct cell-specific expression patterns. For example, endothelial cells highly expressed PECAM1, CAVIN2, and PLPP1; epithelial cells highly expressed ELTD1 and PPAP2A; and mast cells specifically expressed TPSAB1, CPA3, and SLC18A2 (**Figure 1C**). These findings further support the reliability of our cell type annotations.

### 3.2 Expression characteristics of NMRGs

To evaluate nucleotide metabolism in ccRCC at the single-cell level, we divided cells into high-score (top 50%) and low-score (bottom 50%) groups based on the median ssGSEA score of nucleotide metabolism. UMAP visualization revealed differences in cell distribution between the two groups across different clusters (**Figure 1D**), suggesting significant metabolic heterogeneity in nucleotide metabolism among cell subpopulations. Differential expression analysis between the high-score and low-score groups (limma package; |log2FC| > 0.5, P < 0.05) identified 137 significantly upregulated genes in the high-score group (**Figure 1E**).

We then applied five machine learning algorithms—Boruta, XGBoost, LASSO, Random Forest (RF), and Pamr—to screen these 136 differentially expressed genes. Integration of the algorithm outputs identified 127 candidate key genes for subsequent analysis (**Figure 1F**). These genes may play crucial roles in regulating metabolic reprogramming and tumor progression in ccRCC. Detailed gene lists generated by Boruta, XGBoost, LASSO, RF, and Pamr are provided in **Supplementary Table 3**.

### 3.3 Construction of the NMRGs signature

**Figure 2:**
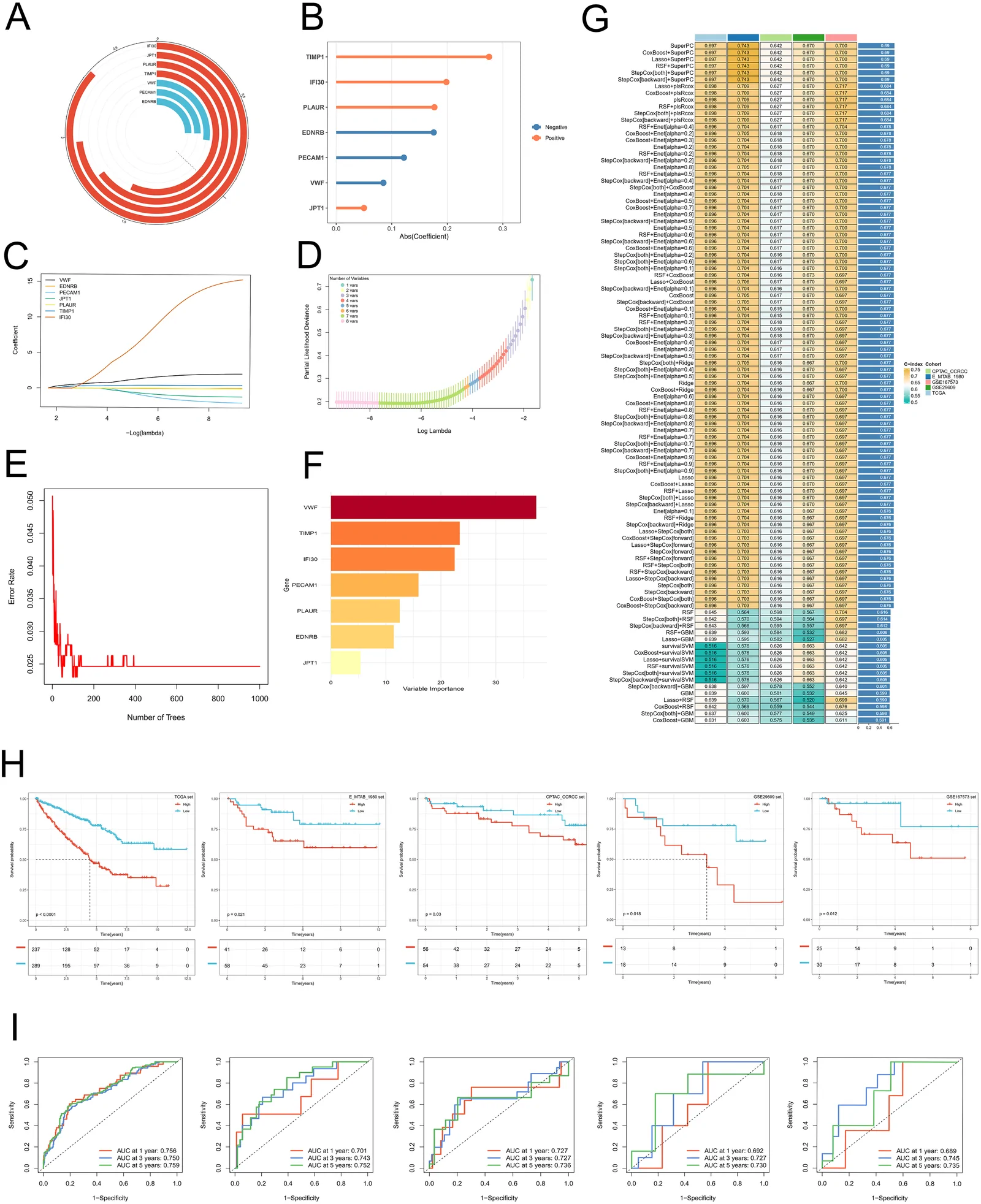
Construction of an ML-based prognostic model for ccRCC. **(A)** Univariate Cox regression analysis. **(B)** Feature selection using CoxBoost. **(C, D)** Feature selection using LASSO. **(E, F)** Feature selection using random forest (RF). **(G)** C-index of 101 ML models across training and validation cohorts. **(H)** Kaplan-Meier curves for overall survival (OS) in high- vs. low-risk groups. **(I)** Time-dependent ROC curves for predicting 1-, 3-, and 5-year OS.

In the TCGA-ccRCC dataset, univariate Cox proportional hazards regression analysis was performed on the 127 candidate genes identified through screening, resulting in the identification of seven key genes significantly associated with patient prognosis: TIMP1, IFI30, PLAUR, EDNRB, PECAM1, VWF, and JPT1 (**Figure 2A**). Detailed univariate Cox regression results for these seven hub genes, including HRs, 95% confidence intervals, and P values, are provided in **Supplementary Table 5**. We then evaluated the predictive performance of these genes using multiple machine learning methods, including CoxBoost (**Figure 2B**), LASSO (**Figures 2C, D**), and Random Forest (RF) (**Figures 2E, F**). These results supported the prognostic value of the seven hub genes in ccRCC. Based on these genes, we constructed the NMRGs signature.

Among the 101 machine-learning algorithm combinations, the SuperPC model showed favorable overall performance across the TCGA-ccRCC training cohort and four independent validation cohorts, including GSE29609, GSE167573, E-MTAB-1980, and CPTAC-ccRCC (**Figure 2G**). Detailed C-index values of the 101 machine-learning model combinations across the TCGA training cohort and four external validation cohorts are provided in **Supplementary Table 6**. Importantly, SuperPC was not selected solely based on the highest C-index in the training cohort, but rather on its overall predictive performance and cross-cohort stability. We further evaluated the prognostic performance of the SuperPC-based NMRGs signature in the TCGA-ccRCC dataset and the four external cohorts. Kaplan–Meier survival analysis showed that patients with high NMRGs scores had significantly poorer overall survival than those with low scores across multiple cohorts (log-rank P < 0.05; **Figure 2H**), suggesting that the model had relatively stable prognostic stratification ability rather than training-set-specific overfitting.

ROC curves further evaluated the predictive capability of the model. In the TCGA-ccRCC cohort, the AUC values of the NMRGs signature for predicting 1-, 3-, and 5-year overall survival were 0.756, 0.750, and 0.759, respectively. The model also demonstrated stable predictive performance across the external validation datasets: GSE29609, GSE167573, E-MTAB-1980, and CPTAC-ccRCC (**Figure 2I**).

### 3.4 Correlation between NMRGs signature and clinical characteristics

**Figure 3:**
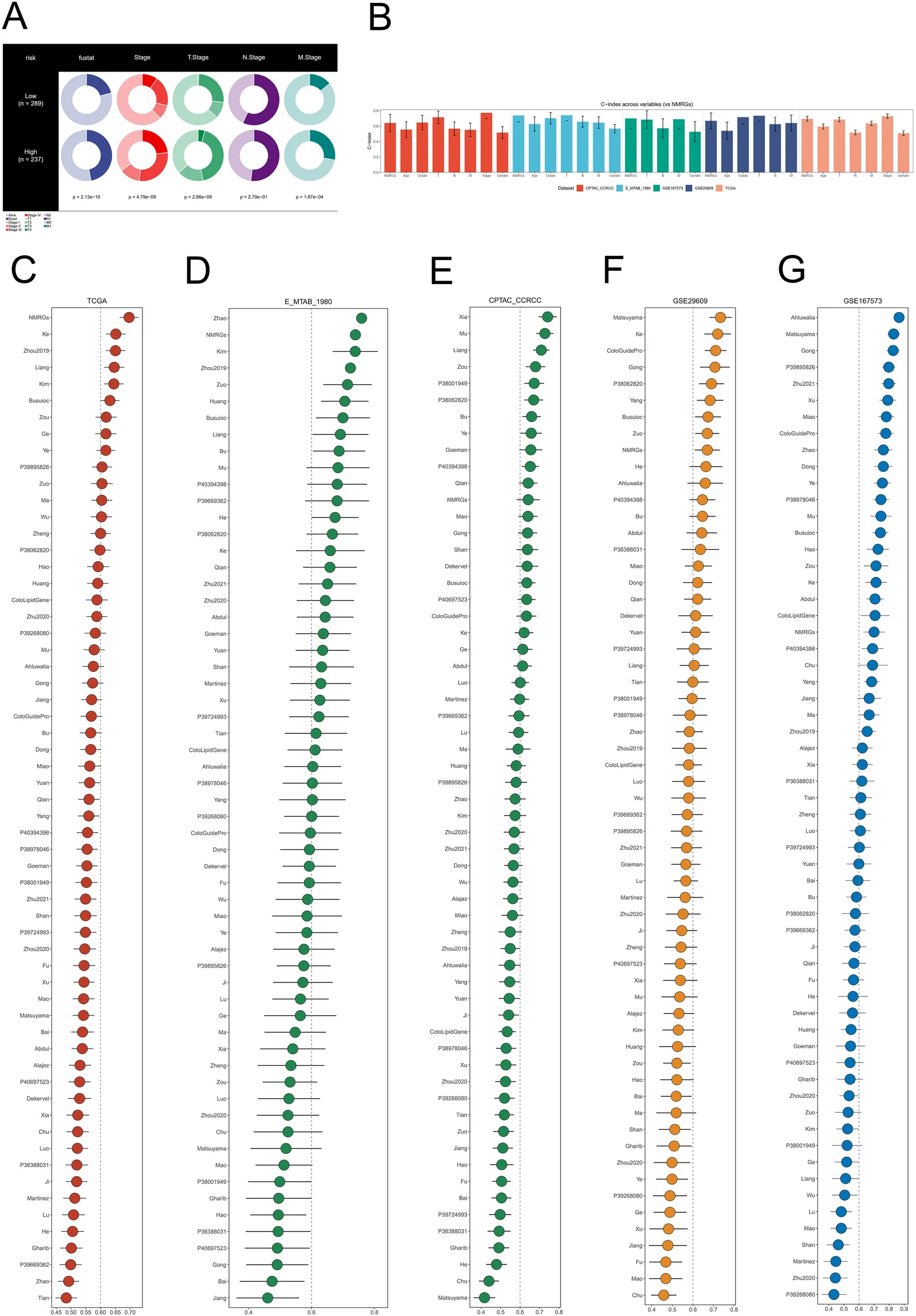
Prognostic value of the NMRG signature. **(A)** Circos plot comparing clinical characteristics between high- and low-risk groups. **(B)** C-index of the NMRG signature and clinical factors across five independent cohorts. **(C–G)** Comparison of C-index between the NMRG signature and 122 published ccRCC prognostic models.

Analysis of the TCGA-ccRCC dataset revealed that the NMRGs signature was significantly correlated with patient survival status, tumor stage, and T and M stages. Specifically, the high-risk group exhibited significantly higher mortality rates and a greater proportion of metastatic patients compared with the low-risk group (P < 0.05; **Figure 3A**).

Further comparative analysis across different external cohorts showed that the NMRGs signature achieved generally higher C-index values than age, tumor stage, and TNM staging, indicating its relatively stable and favorable clinical predictive performance (**Figure 3B**). To further evaluate its predictive performance, we compared the NMRGs signature with 122 previously published prognostic models collected from the literature. The risk scores of these published models were recalculated using the reported gene coefficients and corresponding expression profiles. Detailed information on the reference models, including PMID, model type, gene composition, coefficients, and software/package versions used for recalculation, is provided in **Supplementary Table 4**. The NMRGs signature showed favorable C-index performance compared with most published prognostic models across multiple cohorts (**Figures 3C–G**).

### 3.5 NMRGs signature represents nucleotide metabolic characteristics in ccRCC

**Figure 4:**
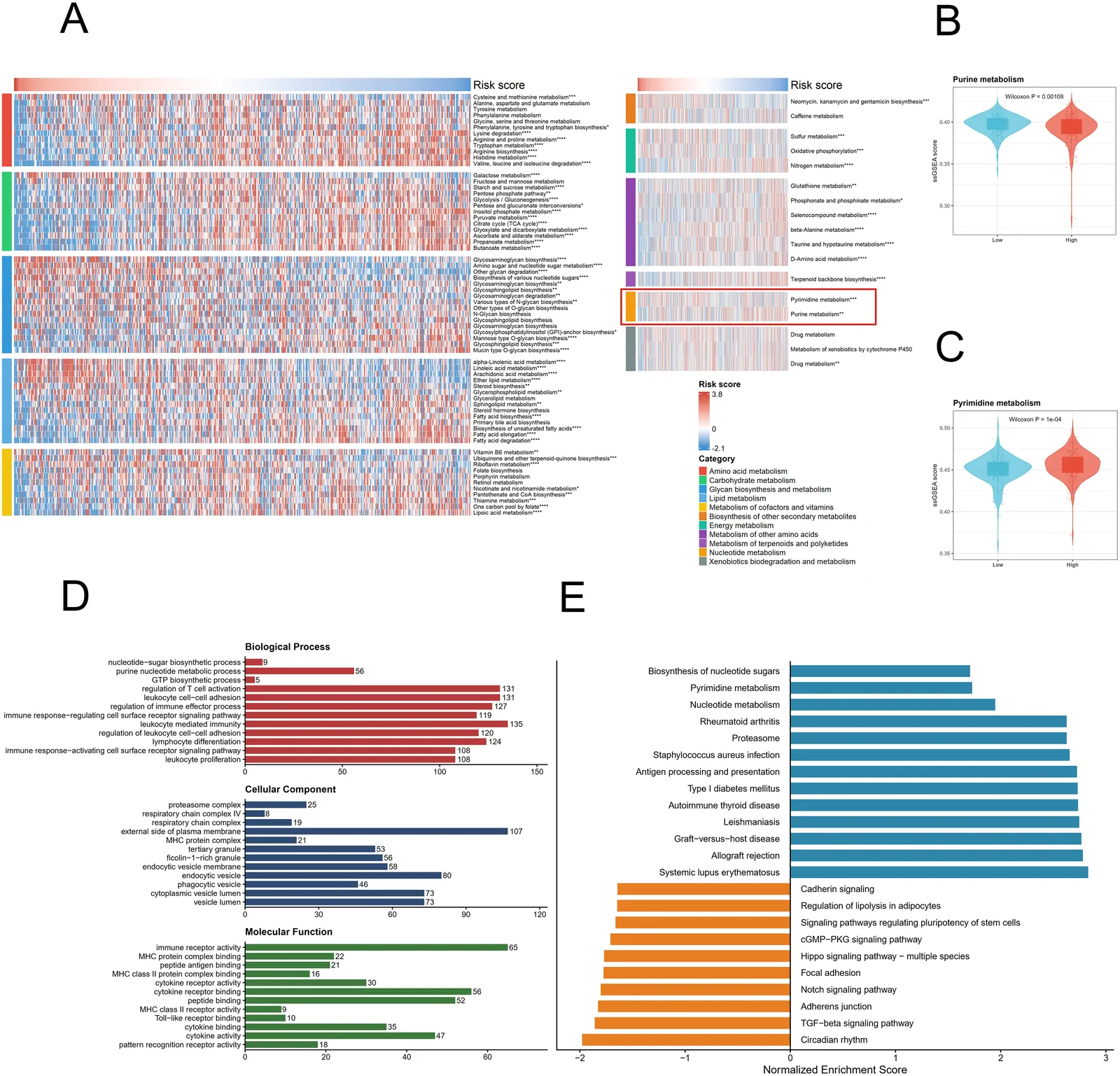
Metabolic characteristics of NMRGs signature. **(A)** Heatmap showing differences in KEGG metabolic pathway activities between high- and low-risk groups. Nucleotide metabolism-related pathways are highlighted by boxes. **(B, C)** Comparison of purine metabolism and pyrimidine metabolism activities between risk groups using the Wilcoxon test. **(D)** GO enrichment analysis of IFI30-associated genes. **(E)** KEGG pathway enrichment analysis of IFI30-associated genes.

To explore the association between the NMRGs signature and metabolic characteristics in ccRCC, we performed ssGSEA analysis based on KEGG metabolic pathways across different risk groups. The analysis covered major metabolic pathways, including amino acid, carbohydrate, lipid, energy, nucleotide, and xenobiotic metabolism. Most pathways showed varying degrees of activation across risk groups (**Figure 4A**). Given the focus of this study on nucleotide metabolism, nucleotide metabolism-related pathways were highlighted in the heatmap. Notably, patients in the high-risk group exhibited higher activity of nucleotide metabolism-associated pathways, particularly purine metabolism and pyrimidine metabolism, which were further analyzed separately (**Figures 4B, C**). These results suggested that the NMRGs signature was closely associated with nucleotide metabolic reprogramming in ccRCC.

Among the seven model genes, IFI30 was consistently selected by multiple algorithms. Its classical function is to promote MHC class II-restricted antigen processing and presentation, thereby activating CD4+ T lymphocyte immune responses (29). However, current research has primarily focused on the immunoregulatory functions of IFI30 (30), while its role in tumor cell metabolic regulation remains unclear, and systematic studies on the association between IFI30 and ccRCC are lacking. Using the LinkedOmics database to analyze IFI30 co-expression patterns in ccRCC (|Statistic| ≥ 0.30, FDR < 0.05), we identified 1,836 positively correlated genes and 1,004 negatively correlated genes. GO enrichment analysis revealed that the IFI30-associated gene network is linked not only to tumor immune activity but also to nucleotide metabolism and related synthetic processes (**Figure 4D**). Further KEGG-GSEA analysis showed positive enrichment of pathways including Nucleotide metabolism, Pyrimidine metabolism, and Biosynthesis of nucleotide sugars. Notably, Nucleotide metabolism, as the core pathway, suggests that IFI30 may be involved in nucleotide metabolism in ccRCC (**Figure 4E**).

### 3.6 IHC in human tissue samples and cell model analysis

**Figure 5:**
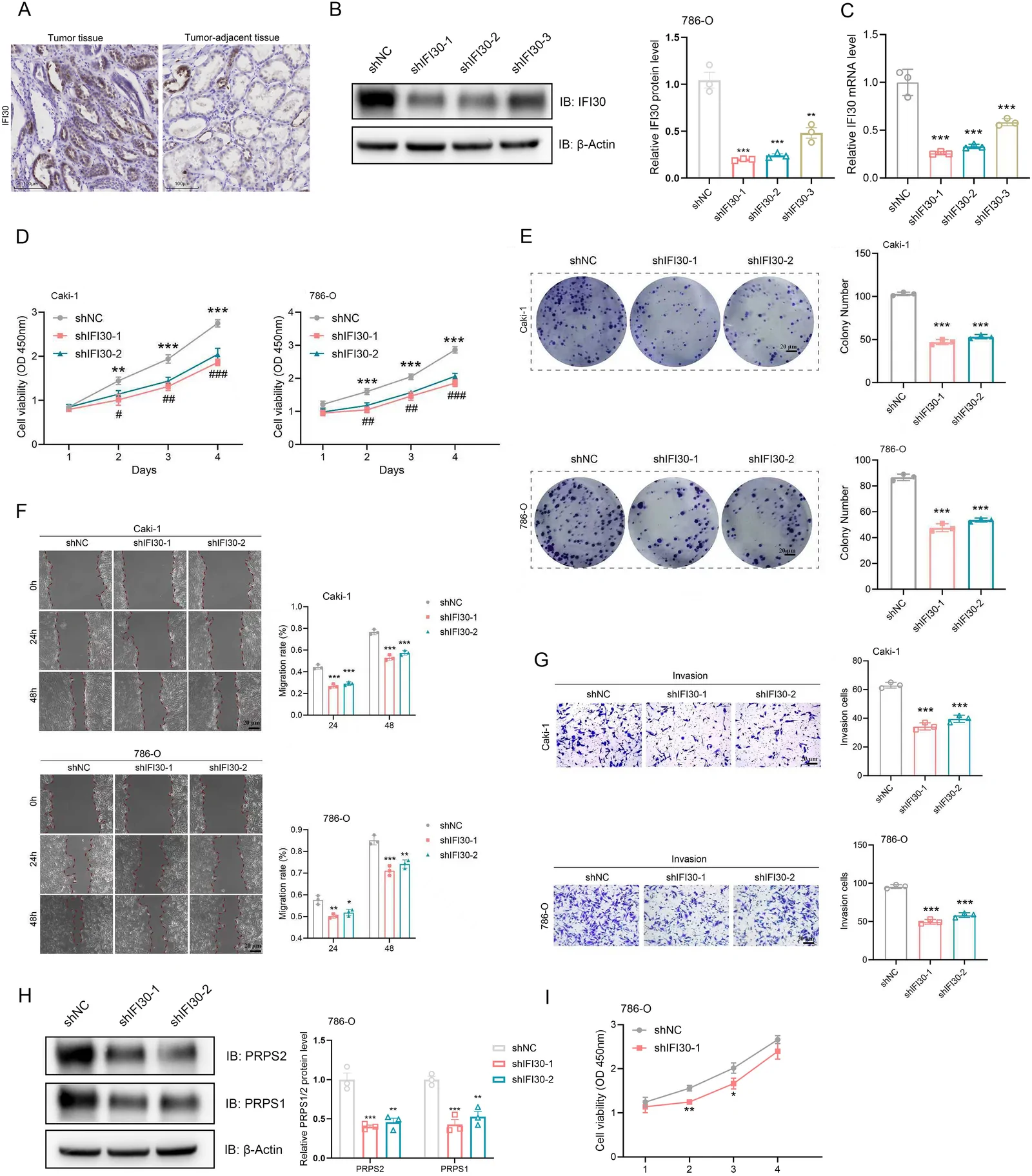
IFI30 promotes ccRCC progression via regulating nucleotide metabolism. **(A)** IHC staining of IFI30 in ccRCC tumor vs. adjacent normal tissues. **(B)** qRT-PCR validation of IFI30 knockdown efficiency in 786-O and Caki-1 cells. **(C)** Western blot validation of IFI30 knockdown efficiency. **(D)** CCK-8 assay for cell proliferation. **(E)** Colony formation assay. **(F)** Wound healing assay. **(G)** Transwell invasion assay. **(H)** PRPS1/2 expression upon IFI30 knockdown by Western blot. **(I)** Nucleotide rescue experiment: 50 μM nucleoside mixture restored cell growth at 72 h. Scale bar: 20 μm. Data are mean ± SD. *p < 0.05, **p < 0.01, ***p < 0.001, ^#^p<0.05, ^##^p<0.01, ^###^p<0.001.

To validate IFI30 expression in ccRCC, we performed IHC on surgical specimens. IFI30 showed strong cytoplasmic staining in tumor cells but weak staining in adjacent normal tissues across all three specimens (**Figure 5A**), confirming its high expression and suggesting a role in ccRCC progression. We then established IFI30-knockdown ccRCC cell models using three shRNA sequences (shIFI30-1, -2, -3) via lentiviral transduction in 786-O and Caki-1 cells. After qRT-PCR and Western blot validation, the two most effective sequences (shIFI30–1 and shIFI30-2) were selected (**Figures 5B, C**). Compared with the control group, PRPS1/2 protein expression levels were significantly downregulated in the IFI30 knockdown group (P < 0.05) (**Figure 5H**). Notably, exogenous supplementation with a 50 μM nucleoside mixture restored cell growth to control levels after 72 hours (P > 0.05) (**Figure 5I**). These results indicate that IFI30 is involved in regulating nucleotide metabolism in ccRCC cells.

### 3.7 IFI30 knockdown suppresses malignancy in ccRCC cells

To investigate the effects of IFI30 knockdown on ccRCC cells, we performed CCK-8, colony formation, wound healing, and Transwell assays.CCK-8 results showed that IFI30 knockdown significantly inhibited the proliferation of 786-O and Caki-1 cells (P < 0.01; **Figure 5D**). The colony formation assay revealed that the shIFI30 group formed significantly fewer colonies than the control group (P < 0.01; **Figure 5E**). In the wound healing assay, the shIFI30 group exhibited significantly lower wound closure rates at 24 and 48 hours post-scratching compared to the control group (P < 0.01; **Figure 5F**). Transwell assays further confirmed that the number of invading cells was significantly reduced in the shIFI30 group (P < 0.01; **Figure 5G**). Collectively, these results indicate that IFI30 plays an oncogenic role in ccRCC, and its knockdown effectively suppresses tumor cell proliferation, migration, invasion, and clonogenicity.

### 3.8 IFI30 knockdown suppresses tumor formation in nude mice

To validate the effects of IFI30 on ccRCC proliferation and apoptosis *in vivo*, we subcutaneously injected BALB/c nude mice with 786-O cells stably transfected with shNC, shIFI30-1, or shIFI30–2 lentiviruses. Tumor volumes were monitored regularly, and mice were euthanized on day 21 post-injection, after which tumors were excised.

**Figure 6:**
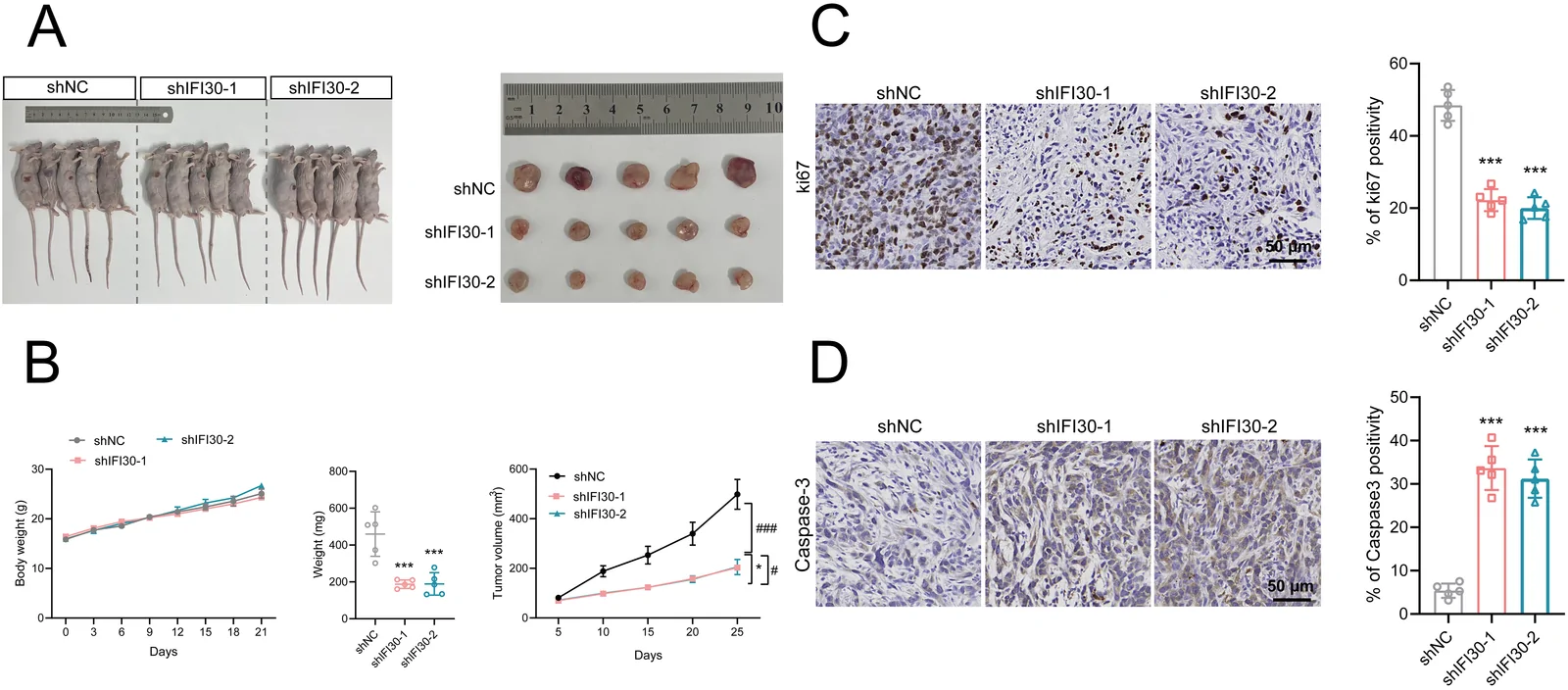
IFI30 knockdown inhibits tumor progression in nude mice. 786-O cells with IFI30 knockdown were injected into nude mice. **(A)** Representative images of tumors and nude mice. **(B)** Body weight, tumor weight, and tumor volume. **(C, D)** Histological analysis of tumor sections by H&E staining and immunohistochemistry for Ki-67 and Caspase-3. Scale bar: 50 μm. Data are presented as mean ± SD. *p < 0.05, ***p < 0.001, ^#^p<0.05, ^###^p<0.001.

Compared with the shNC control group, subcutaneous tumor growth was significantly slowed in mice injected with shIFI30–1 or shIFI30–2 cells. Tumor volume and weight in both shIFI30 groups were significantly lower than those in the shNC group (P < 0.01), with the difference increasing over time. No significant changes in body weight were observed among the groups (P > 0.05; **Figures 6A, B**).Immunohistochemical staining further revealed that the percentage of Ki67-positive cells was significantly reduced in the IFI30-knockdown group compared with the control group (P < 0.01; **Figure 6C**), indicating that IFI30 knockdown inhibited tumor cell proliferation. Concurrently, the percentage of Caspase-3-positive cells was significantly increased in the IFI30-knockdown group (P < 0.01; **Figure 6D**), indicating that IFI30 knockdown promoted tumor cell apoptosis.

Together, these results demonstrate that IFI30 knockdown exerts antitumor effects *in vivo* by inhibiting proliferation and inducing apoptosis.

## 4 Discussion

Nucleotide metabolism is a hallmark of tumor cells, providing essential building blocks for DNA replication and repair, as well as for RNA synthesis and signal transduction (10). Nucleotide synthesis inhibitors were among the earliest anticancer drugs and remain important for treating many cancers (11). ccRCC is a malignancy highly characterized by metabolic reprogramming (31). Gene expression profiling of renal cell carcinoma samples has revealed that high expression of proliferation-related genes, such as RRM2, is associated with shorter patient survival, suggesting a link between ccRCC and nucleotide metabolism (13). As research has advanced, multiple nucleotide metabolism-related targets have been identified in ccRCC. With the rapid advancement of single-cell sequencing technologies, numerous tumor biomarkers and targets have been discovered (16), and an increasing number of diagnostic and prognostic signatures are being developed to predict clinical outcomes and treatment efficacy. Notably, recent applications of scRNA-seq in tumor progression have provided important insights into NMRGs signatures (32). Additionally, machine learning enables the extraction of the most valuable data from these complex datasets (33).

We integrated scRNA-seq and bulk RNA-seq data to identify NMRGs, mitigating cellular heterogeneity interference. Unlike single-algorithm models, we used 101 machine learning algorithms to reduce selection bias. The best-performing NMRGs signature model was identified by C-index and validated by ROC curves. The model includes seven genes: TIMP1, IFI30, PLAUR, EDNRB, PECAM1, VWF, and JPT1. High NMRGs scores correlated with poor overall survival (Kaplan-Meier), as well as with survival status, tumor stage, and T/M stages. This finding suggests that the NMRGs signature may have clinical utility. High-risk patients may be more prone to developing treatment resistance and may therefore require more intensive or alternative therapeutic strategies. Consequently, the NMRGs signature could help guide risk-adapted clinical management of ccRCC. Compared with 122 published risk scores from TCGA-ccRCC, GSE29609, GSE167573, E-MTAB-1980, and CPTAC-ccRCC, our model showed superior accuracy. An ssGSEA scoring system based on KEGG pathways revealed higher nucleotide metabolic activity in high-risk patients, indicating its crucial role in ccRCC progression. Thus, this study is the first to integrate scRNA-seq and bulk RNA-seq data to systematically characterize the nucleotide metabolic heterogeneity landscape of ccRCC and establish a robust prognostic model.

Our NMRG signature comprises seven genes with diverse biological functions. TIMP1 is a matrix metalloproteinase inhibitor involved in extracellular matrix remodeling; elevated TIMP1 expression predicts poor prognosis in multiple cancers, promoting tumor progression and inhibiting apoptosis via the FAK-PI3K/AKT and MAPK pathways in colon cancer (34). PLAUR participates in the protein degradation network surrounding cells and drives multiple malignancy-associated processes, including angiogenesis, differentiation, proliferation, and migration, with high expression correlating with aggressive tumor behavior (35). EDNRB has been implicated in various aspects of tumor progression; beyond its classical role in endothelin signaling, recent studies have identified EDNRB as a specific marker of tumor-associated macrophages (36). PECAM1 encodes a protein critical for primary tumor growth and proliferation, involving angiogenesis, vascular permeability, and extravasation, and has been shown to contribute to the progression of renal, lung, and breast cancers (37). VWF mediates platelet adhesion and angiogenesis; elevated VWF levels have been associated with poor survival in cancer patients (38). JPT1 is a less characterized gene; emerging evidence suggests it may promote cancer metastasis by affecting cytoskeletal stability and microtubule dynamics, and may also be involved in tumor cell proliferation and apoptosis (39). IFI30 traditionally facilitates the processing and presentation of MHC class II-restricted antigens, thereby activating CD4+ T lymphocyte immune responses and participating in tumor immunity (29).

Among these seven genes, IFI30 consistently emerged across multiple algorithmic screenings, prompting us to further explore its role in ccRCC. Previous studies have largely focused on the association between IFI30 and tumor immunity, while its role in the autonomous malignant progression of tumor cells remains systematically understudied. Recent studies have confirmed that IFI30 autonomously promotes the progression of breast cancer and glioma, extending its functions beyond traditional immune regulation (40–42). However, its expression patterns, biological functions, and prognostic significance in ccRCC remain poorly understood. Therefore, IFI30 was selected as a key gene for further investigation.

To preliminarily investigate the potential role of IFI30 in ccRCC, we performed GO and KEGG analyses on IFI30 and its co-expressed genes. The results revealed that IFI30 and its associated genes were significantly enriched in nucleotide metabolism-related pathways. These findings suggest that IFI30 may exert a tumor cell-autonomous oncogenic effect in ccRCC, independent of its immune function, and that this effect is associated with nucleotide metabolism.

To investigate IFI30 expression in ccRCC, we performed IHC staining on 16 pairs of ccRCC and adjacent normal tissues. IFI30 protein was highly expressed in tumor tissues but weakly expressed in adjacent tissues. Based on these findings, we established 786-O and Caki-1 cell lines with stable IFI30 knockdown. Functional assays showed that IFI30 knockdown significantly inhibited the proliferation, migration, and invasion of ccRCC cells *in vitro*, and suppressed tumor growth while inducing apoptosis *in vivo*. Given that GO/KEGG analyses suggested a link between IFI30 and nucleotide metabolism, we examined the effect of IFI30 knockdown on PRPS1/2 expression. PRPS1/2 catalyzes the conversion of ribose-5-phosphate to PRPP, the first and rate-limiting step in purine and pyrimidine synthesis (43, 44). Western blot revealed that IFI30 knockdown significantly downregulated PRPS1/2 expression. Importantly, nucleotide rescue experiments reversed the phenotypic suppression induced by IFI30 knockdown, further confirming that IFI30 promotes ccRCC progression through regulating nucleotide metabolism. This finding extends the traditional understanding of IFI30 as an immune-related gene. The link between IFI30 and nucleotide metabolism may be explained by the fact that both tumor cells and immune cells rely on common nucleotide precursors, such as glucose and glutamine. Through metabolic reprogramming—including aerobic glycolysis and glutamine catabolism—tumor cells significantly upregulate *de novo* nucleotide synthesis pathways to support rapid proliferation, while immune cell function is equally dependent on nucleotide availability.

The NMRGs signature shows strong nucleotide metabolic characteristics and prognostic value, filling a gap in existing biomarkers and demonstrating clinical potential. It generally outperformed 122 previously established models. Although the NMRGs signature holds potential value, several limitations should be acknowledged. First, all datasets were retrospective and derived from public databases, highlighting the need for prospective multicenter validation. Second, although the SuperPC model showed stable performance across multiple cohorts, the retrospective design inevitably raises the possibility of overfitting. Third, the absence of decision curve analysis limits our ability to assess clinical net benefit, as external cohorts lacked consistent treatment decision data and threshold probabilities. Fourth, only IFI30 was experimentally validated among the seven model genes; the functions of the other six genes and their synergy with IFI30 remain unknown, and the relationships between IFI30 and the AKT, ERK, and TGF-β pathways have yet to be explored. Fifth, the inherent complexity of the tumor microenvironment may prevent scRNA-seq from fully capturing ccRCC cell heterogeneity. Finally, while this study provides preliminary evidence linking IFI30 to nucleotide metabolism, the mechanistic crosstalk between its immune and metabolic functions, as well as its potential role in therapy resistance, requires further experimental investigation. Of note, previous studies have shown that IFI30 on tumor endothelial cells promotes macrophage infiltration via the ATF3-CCL5 axis, suggesting a possible interface between nucleotide metabolism and immunity (40). Given the emerging importance of nucleotide metabolism in tumor resistance, IFI30 may represent a promising therapeutic target in ccRCC. Addressing these questions will be a priority in our future research, which will combine multicenter cohort analysis and spatial transcriptomics to further validate the NMRG risk score.

In summary, this study developed an NMRGs signature that outperforms existing models in predicting ccRCC outcomes and identified IFI30 as a key regulator of nucleotide metabolic reprogramming. These findings expand IFI30’s role from immune regulation to metabolic reprogramming, offering new insights for ccRCC subtyping and metabolism-targeted therapies.

## Data Availability

The original contributions presented in the study are publicly available. This data can be founde here:the GEO database (https://www.ncbi.nlm.nih.gov/geo/), the TCGA dataset (https://xenabrowser.net/), the GeneCards database (https://www.genecards.org/), and the LinkedOmics database (https://www.linkedomics.org/). Some of the original contributions presented in the study are included in the article/supplementary materials, further inquiries can be directed to the corresponding author.
