# Supplementary material for: Integrative analysis of scRNA-seq and bulk RNA-seq with machine learning develops a nucleotide metabolism–based prognostic model for ccRCC and reveals the function of IFI30
Source: Front Oncol. 2026 Aug 14;16:1881692. doi: 10.3389/fonc.2026.1881692 (PMC13522744; doi:10.3389/fonc.2026.1881692)
Supplement: Supplementary file 1 [file Image1.pdf]

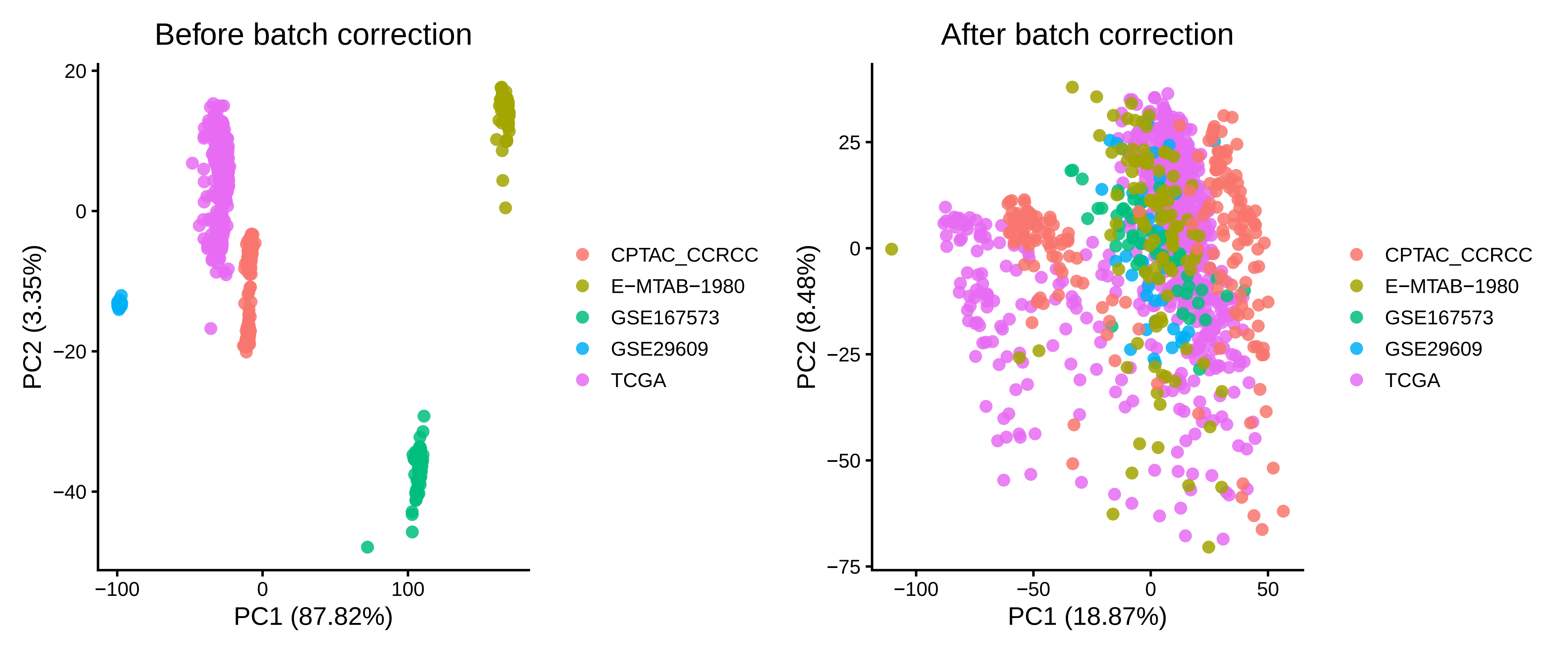
Before batch correction
After batch correction
PC1 (87.82%)
PC2 (3.35%)
PC1 (18.87%)
PC2 (8.48%)
20
0
−20
−40
−100
0
100
25
0
−25
−50
−75
−100
−50
0
50
CPTAC_CCRCC
E−MTAB−1980
GSE167573
GSE29609
TCGA
CPTAC_CCRCC
E−MTAB−1980
GSE167573
GSE29609
TCGA
